# Determination of hematological and biochemical values blood parameters for European bison (*Bison bonasus*)

**DOI:** 10.1371/journal.pone.0303457

**Published:** 2024-05-15

**Authors:** Anna Didkowska, Daniel Klich, Krzysztof Anusz, Marlena Wojciechowska, Marta Kloch, Magdalena Perlińska-Teresiak, Wojciech Bielecki, Wanda Olech

**Affiliations:** 1 Department of Food Hygiene and Public Health Protection, Institute of Veterinary Medicine, Warsaw University of Life Sciences (SGGW), Warsaw, Poland; 2 Department of Animal Genetics and Conservation, Institute of Animal Sciences, University of Life Sciences (SGGW), Warsaw, Poland; 3 Department of Pathology and Veterinary Diagnostics, Institute of Veterinary Medicine, Warsaw University of Life Sciences (SGGW), Warsaw, Poland; Universitat Autonoma de Barcelona, SPAIN

## Abstract

Hematological and biochemical blood parameters are important tools for evaluating animals’ health. They might be crucial in assessing the health of entire populations of wild animals, such as European bison (*Bison bonasus*). The aim of this study was to establish hematological and biochemical values for healthy European bison and to determine whether there were significant relations with age and sex. Blood samples were collected from 79 animals and tested according to generally accepted standards and the results were subjected to statistical analysis. Most of the age and gender-related correlations found in our study were predictable based on previous reports. Due to bone growth, juvenile animals have typically higher ALP and P concentrations relative to adults. Several age-related dependencies were surprising, like higher Na concentration in younger European bison. Determination of hematological and biochemical blood parameters of healthy European bison may significantly contribute to the further restitution of this endangered species.

## Introduction

Biochemical and hematological blood parameters are useful indicators of human and animal health [1–3]. They are helpful for both disease diagnostics in captive animals and health assessment of wildlife populations [4–6]. Values of these parameters vary by species. They have been established for most species and in some cases even breeds [7–10]. Recently, reference values are being established for more and more wildlife species [11–14]. Blood tests can provide crucial information on the health status of endangered wildlife species and therefore can support conservation measures [15].

The European bison (*Bison bonasus*) population struggles with many health threats, including exposure to pesticides [16], mineral deficiencies or heavy metal poisoning [17], and infectious diseases [18]. The Polish population of European bison may be particularly exposed to infectious and invasive pathogens [19] due to its growing density and limited gene pool [20]. Therefore, one of the key goals of restitution is monitoring the health of this endangered species.

In endangered species, where each individual is very valuable, hematological and biochemical tests allow for establishing disease processes and progression, becoming very useful tools for health assessment [13,21]. For this reason, it is useful to define typical values for healthy animals in the case of various environmental or physiological factors [22]. Taking into consideration the limited number of individuals of endangered species it is difficult to obtain reference values of statistical significance [23].

So far, few reports have been published about hematological and biochemical studies on European bison. They were carried out on a limited number of samples [24–27], only during the winter season [28], or involved only a few blood parameters [29,30]. Most animals included in previous research were culled because of suspicion of disease, which may misrepresent the suggested reference value. For this reason, there is still a lack of reliable data on the values of hematological and biochemical parameters for European bison.

Taking into consideration the above, the aim of the present study was to determine the hematological and serum biochemical values in European bison and their relation with sex and age.

## Materials and methods

### Sample material

Blood samples were collected from 79 European bison, including 37 males and 42 females of age 0.5 to 18 years (0.5–18 for males, and 1–16 for females) throughout the year, but mainly during the autumn-winter season (S1 Table). In the case of free-living European bison, age was assessed based on the body mass, and appearance of horns and teeth [31]. Animals were caught in the wild or in enclosures intended for transport, reintroduction to a new location, or collared for spatial distribution monitoring. Pharmacological immobilization was conducted as described previously [32]. Briefly, anesthesia was performed with etorphine hydrochloride (Captivon, 9.8 mg/ml, Wildlife Pharmaceuticals, South Africa) and xylazine (Nerfasin, 100 mg/ml, Livisto, Italy). The average time required after injection of the anesthetic to blood collection was 10 minutes. Vital blood sampling as a veterinary activity during health monitoring does not require the consent of the ethics committee under Polish law. Each of the animals was examined for health-clinical conditions and tested for tuberculosis, bovine leukemia, and brucellosis. Only apparently clinically healthy animals with negative disease results were qualified for this study. Animals usually stayed in given enclosure or were present in given site for a long time before immobilization, at least 6 months. Two animals among the analyzed group died after immobilization (S1 Table).

Blood was collected from the external jugular vein (*vena jugularis externa*) or tail vein (*vena caudalis*). According to the manufacturer’s instructions, blood was collected into 9-ml tubes with a clotting activator (Medlab Products, Raszyn, Poland) for biochemical tests. The serum was obtained after centrifugation (10 min at 2500 g). For hematological tests, blood was collected in test tubes with EDTA-K3 (tripotassium edetate) (Medlab Products, Raszyn, Poland). The samples were delivered to the laboratory under refrigerated conditions. Blood and serum were tested within 24 hours of collection.

### Hematological and serum biochemical analysis

Hematological and biochemical tests were performed at the Department of Laboratory and Clinical Diagnostics of the Department of Clinical Sciences, Faculty of Veterinary Medicine in Warsaw. The analyzers were set up for testing the blood parameters of cattle.

Hematological tests were performed using the Abacus Junior Vet analyzer (Diatron, Stratec Biomedical Systems, Birkenfeld, Germany). The parameters measured were red blood cells (RBCs), hemoglobin (Hb), hematocrit (Hct), mean corpuscular volume (MCV), mean corpuscular Hb (MCH), mean corpuscular Hb concentration (MCHC), platelet (PLT) and white blood cells (WBCs). Differential leukocyte count (neutrophils banded ‐ BAND, neutrophils segmented ‐ SEG, monocytes ‐ MON, eosinophils ‐ EOS, lymphocytes ‐ LYM) was made by Wright-Giemsa-stained blood smear evaluation with a quantitative evaluation of the percentage made in 10 fields. If platelet clumping was present it was noted.

Serum samples were kept at 4°C and analyzed within eight hours of collecting. Hemolyzed or lipemic samples were excluded from the study. Biochemical tests were performed with Miura One Analyser (Pointe Scientific, Warsaw, Poland). The following parameters were determined the activities of aspartate aminotransferase (AST), alanine aminotransferase (ALT), alkaline phosphatase (ALP), and concentration of glucose (Glu), creatinine (CREA), urea (UREA), total protein (TP), calcium (Ca), phosphorus (P), magnesium (Mg), sodium (Na), potassium (K), chlorine (Cl). The readings were performed according to the guidelines of the International Federation of Clinical Chemistry and Laboratory Medicine (IFCC). Briefly, in the determination of AST, the decrease in absorbance measured at a wavelength of 340 nm is proportional to the amount of NAD produced and, therefore, the aminotransferase activity. In the case of ALT, the decrease in absorbance measured at a wavelength of 340 nm is proportional to the amount of NADH produced and, therefore, the aminotransferase activity. In the case of ALP, the rate of p-nitrophenol formation (yellow color) measured at a wavelength of 405 nm is proportional to alkaline phosphatase activity. The concentration of Glu was determined using a peroxidase-phenol-aminophenazone system. The CREA concentration was determined by using a reaction of creatinine with picric acid in an alkaline environment. For CREA an indirect procedure was used in which ammonia formed from urea under the influence of urease was determined. TP was determined based on the reaction with copper ions in an alkaline environment. Arsenazo III was used to determine Ca concentration, and the intensity of the purple color is directly proportional to the calcium concentration. To determine P concentration, the method of forming a phosphomolybdenum complex was used, followed by reduction of the blue-colored complex using surface-active compounds. To access Mg concentration, the fact that magnesium ions in an alkaline environment react with xylidine blue (MAGON) to form a colored light-absorbing complex was used. K, Cl, and Na concentrations were determined on ion-selective electrodes. The final number of samples included in the assessment of each hematological and biochemical parameter can be found in Tables 1 and 2, respectively.

**Table 1:** Hematological parameters of European bison without clinical signs.

Parameter*	Mean	SD**	Median	Min-Max	N†
WBC(x10^12^/L))	6.2	2.1	6.2	1.9–12.7	49
RBC (x10^9^/L)	6.6	1.5	6.4	2.7–11.2	51
Hb (g/dl)	10.5	2.1	10.3	4.3–16.3	49
Hct (L/L)	30.8	5.7	30.9	12.8–47.1	48
MCV (fL)	48.7	5.6	48.0	39.0–64.0	51
MCH (pg)	16.4	1.5	16.4	13.4–19.5	50
MCHC (g/L)	339	14	340	303–374	51
PLT (x10^9^/L)	170.2	81.8	147.5	28.0–380.0	50
BAND (x10^9^/L)	0.036	0.079	0.0	0.0–0.408	49
SEG (x10^9^/L)	3.391	1.430	3.206	0.731–7.262	49
EOS (x10^9^/L)	0.452	0.432	0.317	0–1.748	49
LYM (x10^9^/L)	2.189	0.861	2.028	0.602–4.178	49
MONO (x10^9^/L)	0.080	0.109	0.056	0–0.601	49
BASO (x10^9^/L)	0.034	0.056	0.0	0–0.255	49

*WBC–white blood cells, RBC–red blood cells, Hb–hemoglobin, Hct–hematocrit, MCV–mean corpuscular volume, MCH–mean corpuscular Hb, MCHC–mean corpuscular Hb concentration, PLT–platelets, SEG–neutrophils segmented, BAND–neutrophils banded, MON–monocytes, EOS–eosinophils, LYM–lymphocytes

** SD–standard deviation

† N–number of samples.

**Table 2:** Biochemical parameters of European bison without clinical signs.

Parameter*	Mean	SD**	Median	Min-Max	N †
AST (U/L)	71.6	15.6	73.2	23.0–115.3	72
ALT (U/L)	25.9	7.0	25.9	10.4–44.7	79
ALP (U/L)	48.0	12.4	45.4	23.5–85.6	73
Glu (mmol/L)	5.64	2.96	5.09	0.48–14.67	77
CREA (mg/%)	1.8	0.5	1.8	0.5–3.2	78
UREA (mmol/L)	6.30	2.06	6.57	2.22–11.42	78
TP (g/L)	60.0	6.5	59.8	40.9–77.6	79
Ca (mmol/L)	2.33	0.24	2.37	1.84–2.93	79
P (mmol/L)	1.47	0.43	1.42	0.42–2.40	78
Mg (mmol/L)	0.85	0.20	0.85	0.42–1.46	78
K (mmol/L)	50.3	10.2	48.7	33.6–81.0	78
Na (mmol/L)	1364	35.4	1369	1297–1458	78
Cl (mmol/L)	1013	40.7	1005	934–1116	69

* AST–aspartate aminotransferase, ALT–alanine aminotransferase, ALP–alkaline phosphatase, Glu–glucose, CREA–creatinine, UREA–urea, TP–total protein, Ca–calcium, P–phosphorus, Mg–magnesium, Na–sodium, K–potassium, and Cl–chlorine

** SD–standard deviation

† N–number of samples.

### Statistical analysis

Before the statistical analysis, we used a modified Z-score method (based on Median Absolute Deviation) to detect outliers for each of the blood parameters [33]. After removing outliers, the median and range for each parameter were calculated for the entire dataset and broken down by sex of the animals. Then, each parameter was compared in terms of sex and age. Individuals up to two years of age were treated as juveniles, and individuals three years and older were placed in the group of adults [34]. In the analysis, all continuous variables were checked whether they were close to the normal distribution, then the Analysis of Variance was performed, taking into account two factors: sex and age of the animals. In the next step, where a statistically significant effect of sex or age was found, a pairwise comparison of the marginal means with the Bonferroni correction was performed (Count variables: MCV, PLT, BAND, LYM, MON, BASO). Differential leukocyte count, was compared using a generalized linear model with a Poisson distribution and a log link function. Two factors were also included in the model: sex and age of the animals. If in a given blood parameter a significant effect of sex or age was found, a pairwise comparison of the marginal means with the Bonferroni correction was performed.

We also visualized the distributions of blood parameters separately for sex of animals. The visualizations were performed using R Statistical Software (v4.2.1) [35] with ggplot2 (v3.4.1) [36], greasy (v0.1.4) [37], and grudges (v0.5.4) [38] packages. Data preparation for visualization was performed using readxl (v1.4.2) [39], devtools (v2.4.5) [40] and dplyr (v1.1.0) [41] packages.

## Results

Median, SD, reference, and range values for each parameter for the entire population are shown for hematology parameters in Table 1 and biochemical parameters in Table 2.

Out of 14 analyzed European bison blood parameters, only six (RBC, Hb, Hct, BAND, EOS and BASO) showed no statistically significant differences in terms of sex or age (S2A Table). Some parameters showed differences in age only (WBC, MCV, MHC, MCHC, SEG, LYM MONO), and only platelets differed with regard to both sex and age. Juveniles of European bison showed higher than adults’ counts of WBC (marginal mean equaled 7.23 and 5.25 x10^9^/L, respectively), MCHC (marginal mean equaled 344 and 334 g/L, respectively), and almost twice as high count of MONO (marginal mean equaled 0.040 and 0.016 x10^9^/L, respectively). Juveniles showed lower than adults’ counts of MCV (marginal mean equaled 46.3 and 50.8 fL, respectively), MCH (marginal mean equaled 15.9 and 16.8 pg, respectively) and PLT (marginal mean equaled 164.0 and 171.9 x10^9^/L, respectively) but higher than adults counts of SEG (marginal mean equaled 4.05 and 2.74 x10^9^/L), LYM (marginal mean equaled 2.51 and 1.95 x10^9^/L) and MONO (marginal mean equaled 0.12 and 0.04 x10^9^/L). Females of European bison showed lower than males’ counts of PLT (marginal mean equaled 151.8 and 185.7 x10^9^/L, respectively).

**Fig 1:**
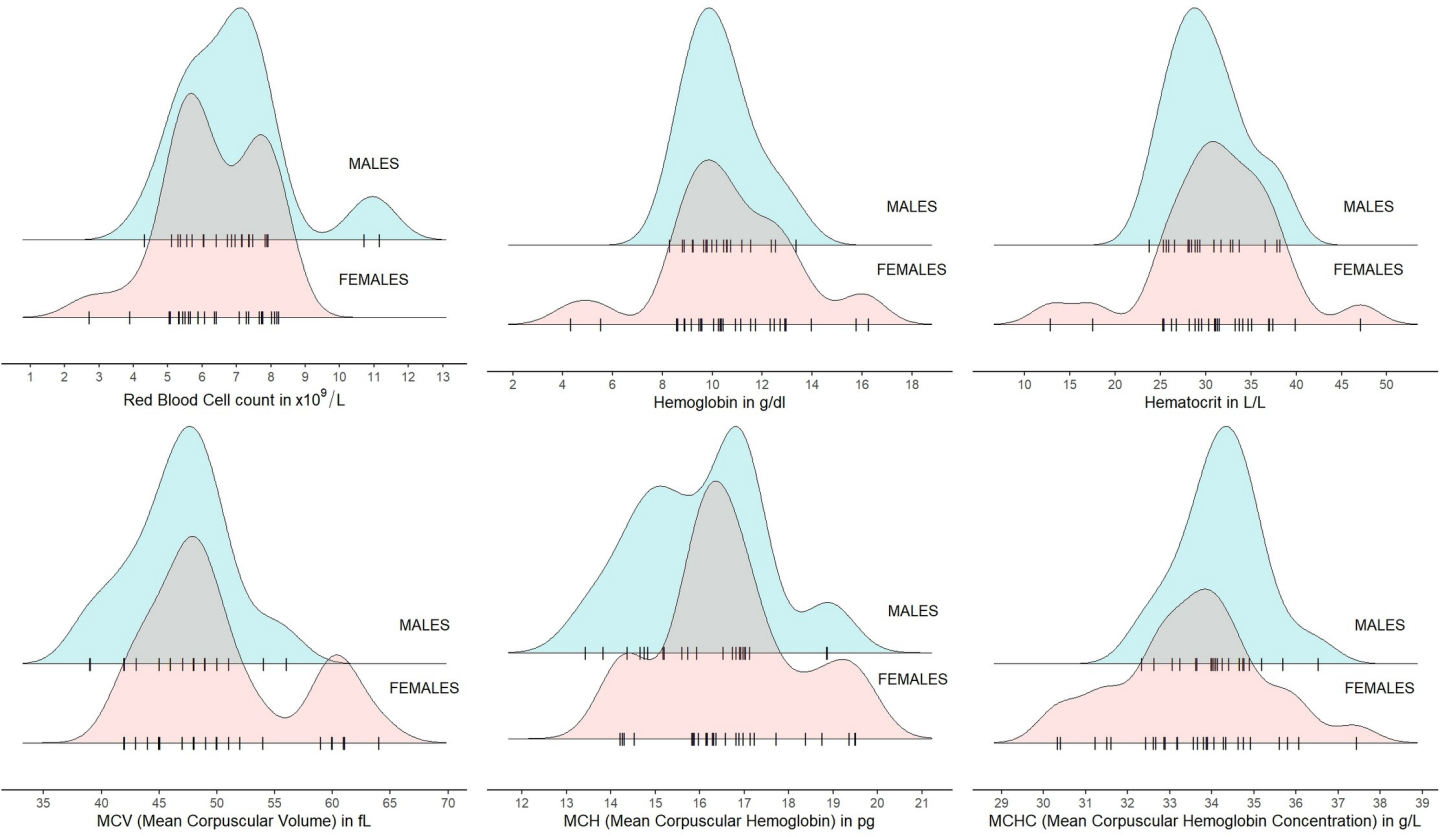
Distribution of red blood cell parameters (WBC, RBC, Hb, Hct, MCV, MCH, and MCHC) in blood of European bison by sex. Jitter points (individual animals) for each sex were marked with the symbol "|" (raw data in S2B Table).

The distribution of red blood cell parameters was similar in both sexes and generally values around the mean dominated. Females for Hb, Hct, MCV and MCHC showed more extreme values than males (Fig 1). In the case of RBC, some males presented higher values. Inverse relation was observed in the MHC (Fig 1).

**Fig 2:**
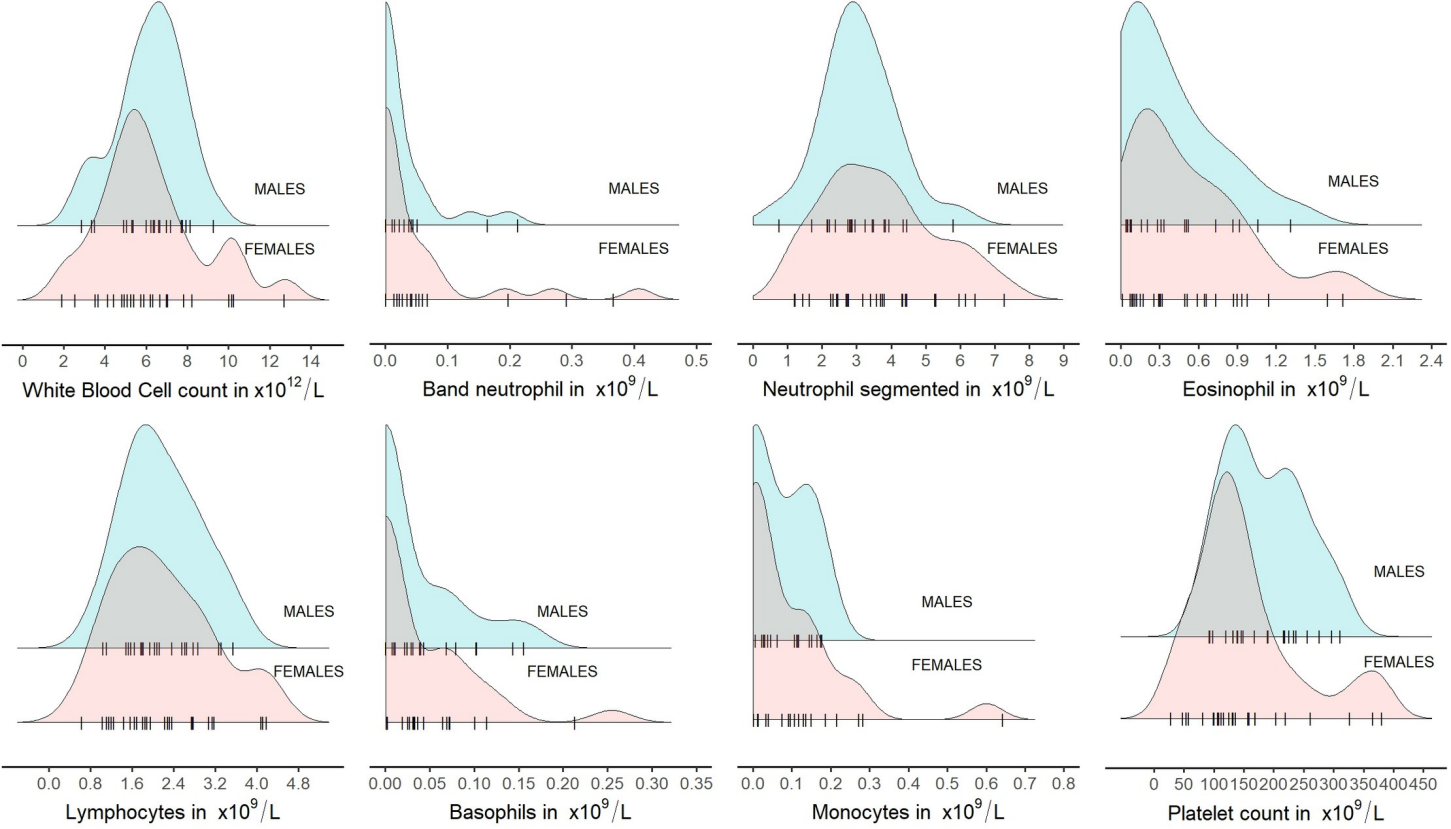
Distribution of white blood cells parameters (BAND, EOS, BASO, MONO, WBC, SEG, and LYM) and platelets (PLT) in blood of European bison by sex. Jitter points (individual animals) for each sex were marked with the symbol "|" (raw data in S2B Table).

The distributions of white blood cell parameters were more varied. Only for WBC, SEG, and LYM, values tend to cluster around the mean with single cases of males or females with extreme values (Fig 2). Other parameters of white blood cells (BAND, EOS, BASO, MONO) presented right-skew distributions, where values close to zero dominate. Usually, there were extreme high values for both sexes (Fig 2). In the case of PLT, males presented a distribution close to normal, while the values in females clustered below the average with simultaneous higher values of some individuals (Fig 2).

Of the 13 biochemical parameters analyzed, seven (AST, ALT, Glu, UREA, Ca, Mg and Cl) showed no statistical differences between sex and age (S3A Table). The others showed differences in age only (CREA, TP, Na), sex only (K), or both, sex and age (ALP and P). Juvenile European bison showed higher than adult activities of ALP (marginal mean equaled 52.3 and 43.8 U/l, respectively) and concentrations of P (marginal mean equaled 1.626 and 1.325 mmol/L, respectively), but lower concentrations of CREA (marginal mean equaled 1.73 and 1.96 mg/%, respectively), TP (marginal mean equaled 57.4 and 62.7 g/L, respectively), and Na (marginal mean equaled 1355.7 and 1372.6 mmol/L, respectively). Females presented lower concentrations and activities of all the parameters than males, i.e.: ALP (marginal mean equaled 44.4 and 52.0 U/I, respectively), P (marginal mean equaled 1.32 and 1.63 mmol/L, respectively) and K (marginal mean equaled 47.97 and 52.52 mmol/L, respectively) (S3A Table).

**Fig 3:**
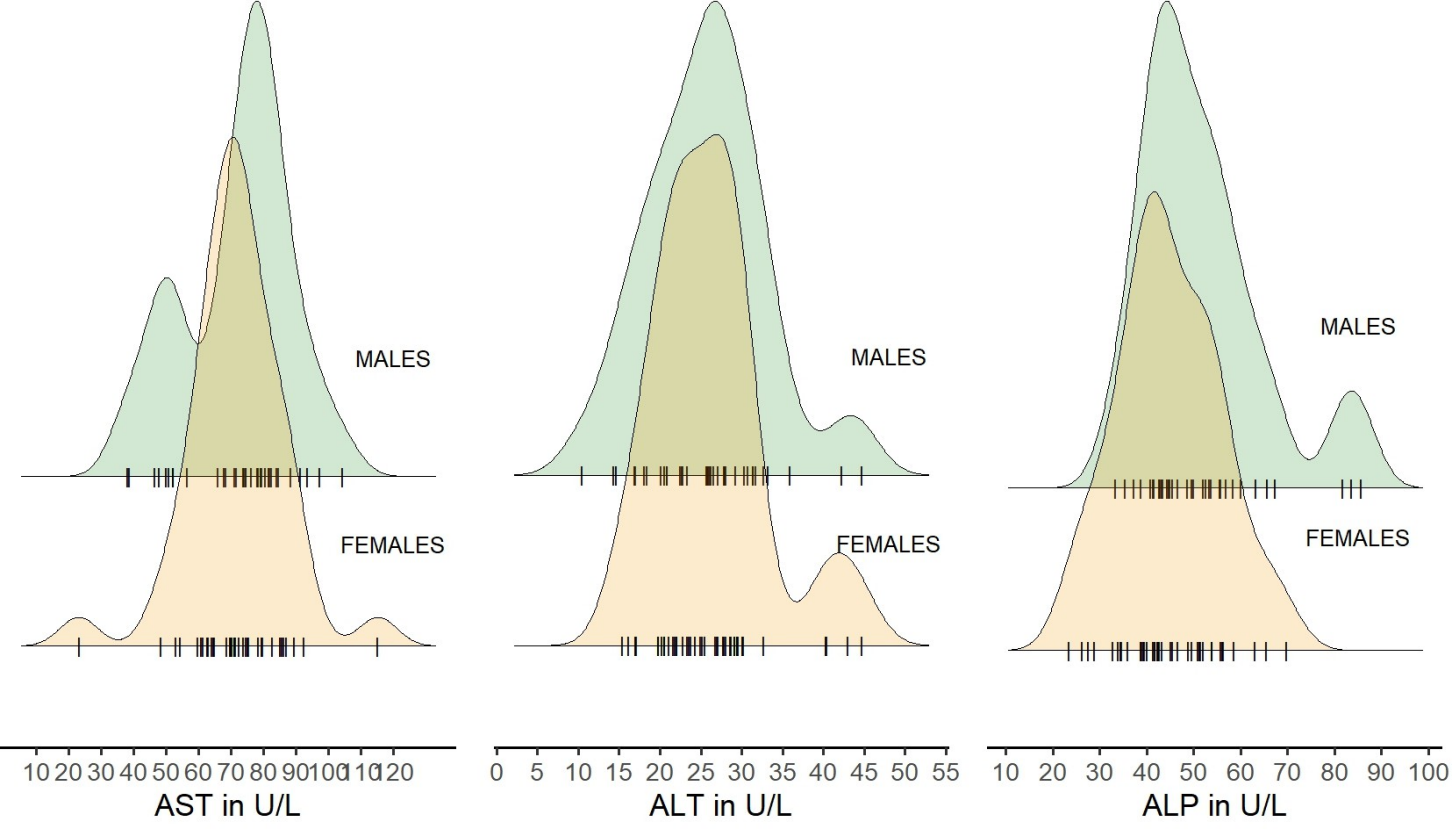
Distribution of liver enzymes (AST, ALT and ALP) in blood of European bison by sex. Jitter points (individual animals) for each sex were marked with the symbol "|" (raw data in S3B Table).

Distribution of liver enzymes (AST, ALT and ALP) were generally similar in both sexes, although there were some extreme values in both sexes (Fig 3).

**Fig 4:**
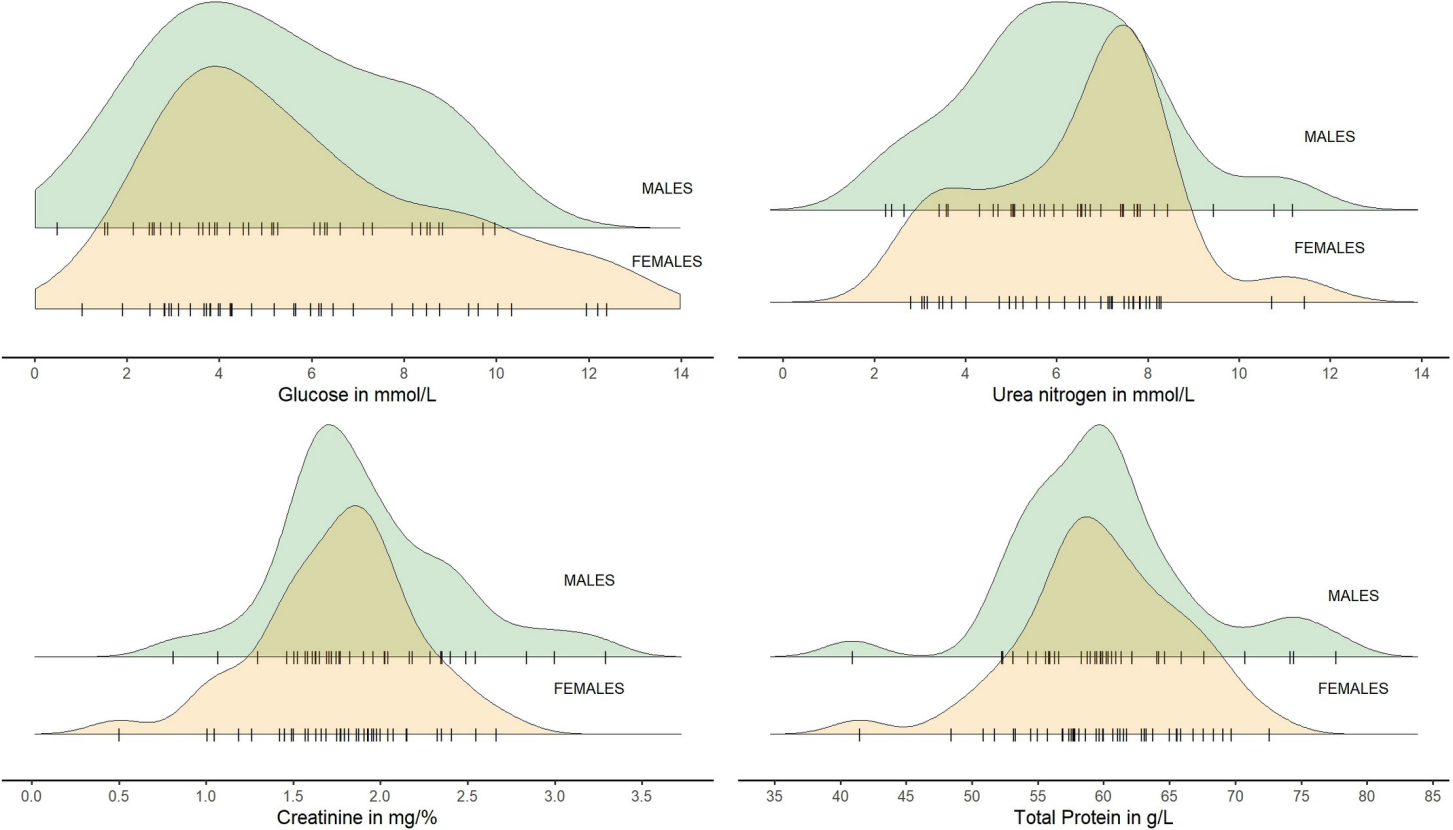
Distribution of Glu, CREA, UREA and TP in blood of European bison by sex. Jitter points (individual animals) for each sex were marked with the symbol "|" (raw data in S3B Table).

The Glu values for females and males were usually similar, although some females presented significantly higher values than males (Fig 4). For CREA and TP, distributions were close to normal and similar for both sexes. Males had extreme higher and lower values, and females only lower extreme values (Fig 4). For UREA, males presented a normal distribution, and females’ values were usually above the mean. Extreme values were observed for both sexes (Fig 4).

**Fig 5:**
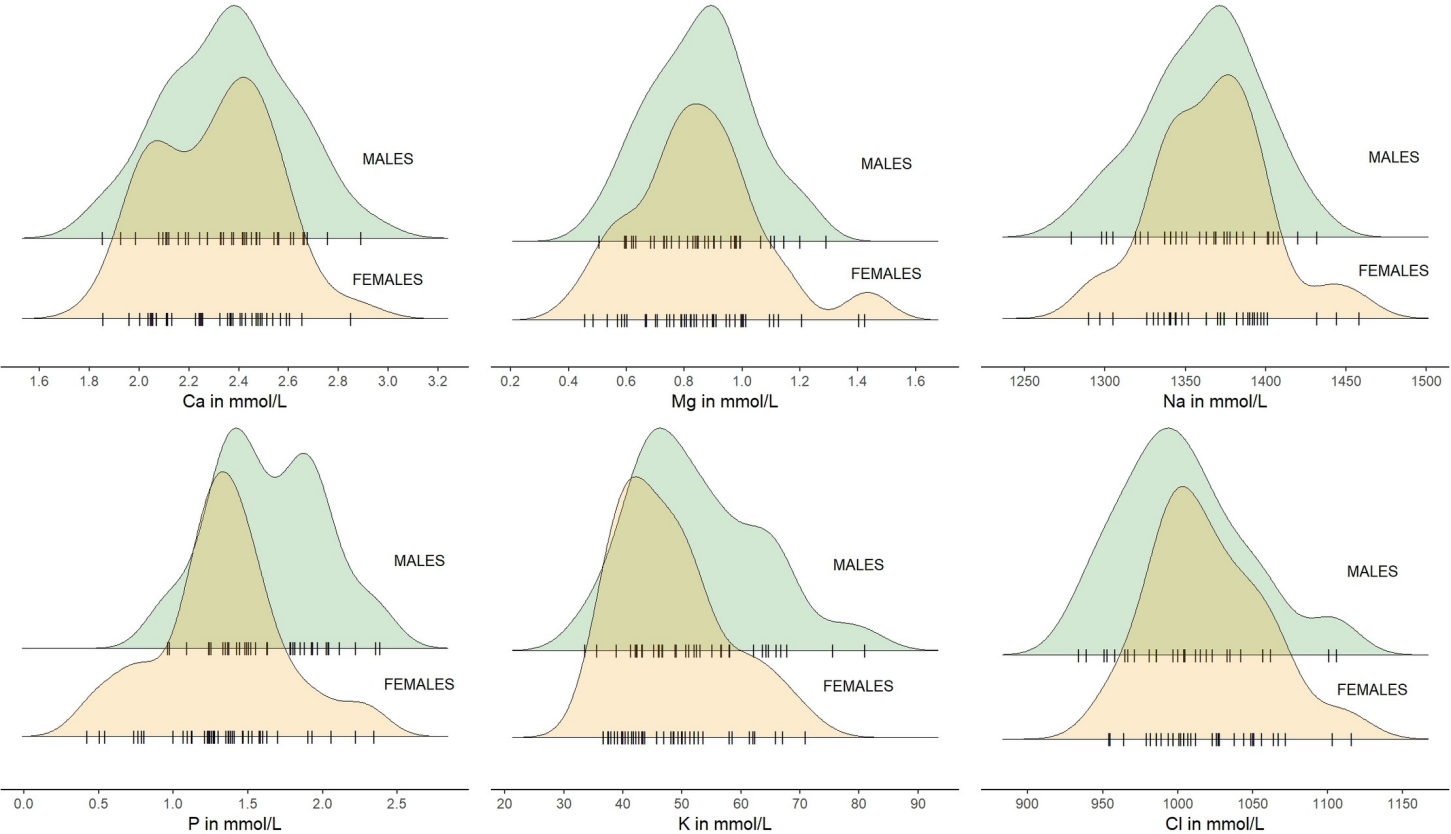
Distribution of the concentration of elements (Ca, Mg, Na, P, K and Cl) in blood of European bison by sex. Jitter points (individual animals) for each sex were marked with the symbol "|" (raw data in S3B Table).

The distribution of the concentration of all studied elements (Ca, Mg, Na, P, K and Cl) was close to the normal distribution and generally similar for both sexes (Fig 5). In females, higher and lower extreme values were usually observed, except from K and Cl. In males, usually higher values in some individuals were usually observed (for Mg, P, K and Cl), except from Na (where lower values were observed) and Ca (where both lower and higher values were observed) (Fig 5).

## Discussion

This study establishes baseline parameters for selected hematologic and biochemical markers in European bison. It will provide a benchmark for veterinary and conservation research on this species. Our study is based on a relatively large sample size (49 to 79 for each parameter) considering the status of the species and numerous health threats.

Previously, studies in this field of veterinary diagnostics were conducted on European bison, but they could not determine the wide spectrum of blood parameters for healthy animals. Wołk et al. [24] tested 46 European bison for hematological values; however, it was conducted only on European bison from Białowieska Forest, and for 12–82% of animals (depending on age group), invasive diseases were confirmed. However, the obtained results were quite similar to those obtained in our study. The largest difference was noted in WBC, where the mean value was 4.73 to 4.82 (depending on the age group), and in our study, the mean value was 6.2. A significantly lower count of eosinophils was also noted in our study, which may be due to the fact that Wołk et al. [24] also included individuals with parasitic infestations. The influence of parasitic invasion on blood parameters has been described in the case of *Ashworthius sidemi* in European bison. A high infection level of *A*. *sidemi* caused the deterioration of red blood cell parameters [42]. Gill et al. [29] aimed at verifying seasonal changes in the red blood cell system in European bison. Groups, depending on age and sex, were tested and the seasonal cyclicity was found only in RBCs diameters in all groups. In our study, the periodicity of the seasons was not determined, samples were collected throughout the year but mostly during autumn and winter. Anusz et al. [28], compared to our results, obtained noticeably higher medians for RBC, WBC, LYM, AST, ALP, TP and UREA. These are typical indicators that are elevated in infectious and invasive diseases [e.g. 43,44].

In the work of Lazar et al. [27] based on a few samples the counts of RBC, Hematocrit, MCH and MCHC were higher than those obtained in our study. The reason for this could be because the analyzed animals were kept at mountainous area which is well known adaptive mechanism [45–47].

American bison (*Bison bison*), a related species to the European bison, were much more often studied with regard to hematology and biochemistry parameters [48–51]. However, due to different climatic conditions and methods, we could not reliably compare the results of these two species. Only one study on biochemistry parameters was done on American bison kept on a farm in Poland, but that study was based on a small number of samples [52].

In our study, some hematological parameters differed in the case of age and sex. Young European bison were characterized by a higher count of WBC than adults, which is consistent with previous studies in wild ruminants and is a natural age-related finding [24,53]. Juveniles showed lower counts of MCV and MCH, which is similar to previous studies [24], as well as lower PLT and EOS than adults. Higher counts of EOS in older individuals may be associated with their longer exposure to parasitic invasions. In our study, younger European bison were characterized by a higher count of MCHC, and MONO than adults, which is not a very common trend in other species. In the study based on a small number of samples, the MCHC of European bison was slightly lower than that of deer and cattle [26]. Females were characterized by a lower count of PLT than males which is contrary to human studies [54].

Many biochemical parameters varied by European bison age. Young animals showed increased ALP activity and P concentrations than adults. This is not surprising as elevated activities and concentrations of these analytics are known to be increased in young animals due to active bone growth and remodeling, as well as circulating growth hormone [55,56]. Similar findings have been reported in many mammal species [57–60]. The decreased TP concentrations reported in juvenile might be related to the developing immune system and has been confirmed in other species as well [61]. Young animals showed decreased CREA which can be correlated with lower muscle mass [62]. Our study revealed the decreased concentration of Na in younger animals, which can be connected with increased Na intake due to the intensive growth, as sodium is an important growth factor for stimulating protein synthesis [63]. Females showed an increased activity of ALP, and concentrations of P and K in comparison to males, which is contrary to previous reports in other species where sex has not influenced those parameters [64]. Dębska et al. [30] found concentration of K much higher than in our study, but study concerned only males from Białowieska Forest.

Our study has several limitations. Even though the sample size of European bison was enough to determine reference values, it was not reaching the recommended number of samples to divide normal intervals for sex and age groups of animals [65]. We however were unable to meet the Quality Assurance and Laboratory Standards Committee (QALS) Guidelines for the Determination of Reference Intervals. Also, some subclinically infected animals affect blood parameters; however, subclinical infections, should not significantly change alert host physiology. Besides, in this study we removed outliers with a modified Z-score method to eliminate possible subclinically infected animals, or animals with undetected infections. In addition, our study did not take into account the possible pregnancy in cows, which could have caused a change in some blood parameters. Another limitation can be the effect of anesthesia on blood cell counts and plasma biochemical values which has been described in other species [66–68]. Despite the above limitation, our study presents blood parameters carried out for the first time on a group of healthy animals, using robust analytical methods, which provides a basis for their use.

The vulnerability of European bison makes it necessary to monitor the population in order to continuously improve and update conservation plans. Having typical values for hematological and biochemical blood parameters for European bison could be one of the important diagnostic tools to achieve it. Most of the age and gender-related changes found in our study were predictable based on previous reports. However, several age-related differences were surprising, e.g., higher Na concentration in younger European bison.

## Conclusions

A large number of blood samples from clinically healthy European bison were analyzed. Hematologic and biochemical parameters were established. The influence of age and sex was determined for individual parameters. Our results can provide an important tool for assessing herd health and help to design management strategies as well as to evaluate the health of individual European bison.

## Supporting information

S1 Table Basic information about the European bison and their maintenance conditions. *animal died after immobilization, Age–age of animal (in years), Month–month of sample collection, Area–enclosure size (in ha).
(DOCX)

S2 Table Effect of age and sex on hematology blood parameters of European bison without diagnosed disease symptoms and raw data on hematology blood parameters of European bison used in Figs 1 and 2. (DOCX)

S3 Table Effect of age and sex on biochemical blood parameters of European bison without diagnosed disease symptoms in analysis of variance and raw data on biochemical blood parameters of European bison used in Figs 3–5. (DOCX)
