# Supplementary material for: Determination of hematological and biochemical values blood parameters for European bison (Bison bonasus)
Source: PLoS One. 2024 May 15;19(5):e0303457. doi: 10.1371/journal.pone.0303457 (PMC11095690; doi:10.1371/journal.pone.0303457)
Supplement: S1 Table — *animal died after immobilization, Age–age of animal (in years), Month–month of sample collection, Area–enclosure size (in ha). (DOCX) [file pone.0303457.s001.docx]

S1 Table. Basic information about the European bison and their maintenance conditions. *animal died after immobilization, Age – age of animal (in years), Month – month of sample collection, Area – enclosure size (in ha).

| ID* | Sex | Age | Conditions | Habitats | Month | Area |
| --- | --- | --- | --- | --- | --- | --- |
| 1 | F | 5 | Free roaming | Carpathian beech forest, meadows | March | NA |
| 2* | F | 16 | Free roaming | Carpathian beech forest, meadows | March | NA |
| 3 | F | 10 | Free roaming | Carpathian beech forest, meadows | March | NA |
| 4 | F | 11 | Free roaming | Carpathian beech forest, meadows | March | NA |
| 5 | M | 14 | Free roaming | lowland deciduous and mixed forests, meadows | December | NA |
| 6 | M | 10 | Free roaming | lowland deciduous and mixed forests, meadows | February | NA |
| 7 | F | 7 | Free roaming | lowland deciduous and mixed forests, meadows | March | NA |
| 8 | M | 18 | Free roaming | lowland deciduous and mixed forests, meadows | March | NA |
| 9 | M | 0.5 | Free roaming | lowland deciduous and mixed forests, meadows | December | NA |
| 10 | F | 8 | Free roaming | lowland deciduous and mixed forests, meadows | December | NA |
| 11 | F | 9 | Free roaming | lowland deciduous and mixed forests, meadows | December | NA |
| 12 | M | 3 | Free roaming | lowland deciduous and mixed forests, meadows | December | NA |
| 13 | F | 3 | Free roaming | lowland deciduous and mixed forests, meadows | December | NA |
| 14 | F | 4 | Free roaming | lowland deciduous and mixed forests, meadows | December | NA |
| 15 | F | 7 | Free roaming | lowland deciduous and mixed forests, meadows | February | NA |
| 16 | M | 0.5 | Free roaming | lowland deciduous and mixed forests, meadows | February | NA |
| 17 | F | 4 | Free roaming | lowland deciduous and mixed forests, meadows | February | NA |
| 18 | F | 1.5 | Free roaming | lowland deciduous and mixed forests, meadows | February | NA |
| 19 | M | 5 | Free roaming | lowland mixed and coniferous forests, meadows | February | NA |
| 20 | F | 9 | Free roaming | lowland mixed and coniferous forests, meadows | February | NA |
| 21 | F | 7 | Free roaming | lowland mixed and coniferous forests, meadows | March | NA |
| 22 | M | 8 | Free roaming | lowland mixed and coniferous forests, meadows | August | NA |
| 23 | M | 3 | Enclosure | meadow, single trees and groves | October | 7 |
| 24 | M | 3 | Enclosure | meadow, single trees and groves | October | 7 |
| 25 | F | 2 | Enclosure | meadow, single trees and groves | October | 7 |
| 26 | M | 2 | Enclosure | meadow, single trees and groves | March | 7 |
| 27 | M | 2 | Enclosure | meadow, single trees and groves | March | 7 |
| 28 | F | 1.5 | Enclosure | meadow, single trees and groves | March | 7 |
| 29 | M | 4 | Enclosure | lowland mixed and coniferous forests, meadows | December | 750 |
| 30 | M | 4 | Enclosure | lowland mixed and coniferous forests, meadows | December | 750 |
| 31 | M | 3 | Enclosure | lowland mixed and coniferous forests, meadows | December | 750 |
| 32 | F | 8 | Enclosure | lowland mixed and coniferous forests, meadows | November | 750 |
| 33 | M | 2 | Enclosure | lowland mixed and coniferous forests, meadows | November | 750 |
| 34 | M | 1 | Enclosure | lowland mixed and coniferous forests, meadows | November | 750 |
| 35 | F | 7 | Enclosure | lowland mixed and coniferous forests, meadows | October | 750 |
| 36 | F | 3 | Enclosure | lowland mixed and coniferous forests, meadows | October | 750 |
| 37 | M | 4 | Enclosure | lowland mixed and coniferous forests, meadows | October | 750 |
| 38 | F | 2 | Enclosure | lowland mixed and coniferous forests, meadows | October | 750 |
| 39 | F | 1 | Enclosure | lowland mixed and coniferous forests, meadows | October | 750 |
| 40 | F | 1 | Enclosure | lowland mixed and coniferous forests, meadows | October | 750 |
| 41 | F | 1 | Enclosure | lowland mixed and coniferous forests, meadows | October | 750 |
| 42 | F | 1 | Enclosure | lowland mixed and coniferous forests, meadows | October | 750 |
| 43 | M | 1 | Enclosure | lowland mixed and coniferous forests, meadows | October | 750 |
| 44 | M | 4 | Enclosure | lowland mixed and coniferous forests, meadows | October | 750 |
| 45 | F | 2 | Enclosure | lowland mixed and coniferous forests, meadows | October | 750 |
| 46 | F | 8 | Enclosure | lowland mixed and coniferous forests, meadows | August | 750 |
| 47 | F | 6 | Enclosure | lowland mixed and coniferous forests, meadows | August | 750 |
| 48 | F | 1 | Enclosure | lowland mixed and coniferous forests, meadows | August | 750 |
| 49 | F | 1 | Enclosure | lowland mixed and coniferous forests, meadows | August | 750 |
| 50 | M | 1 | Enclosure | lowland mixed and coniferous forests, meadows | August | 750 |
| 51 | F | 1 | Enclosure | lowland mixed and coniferous forests, meadows | August | 750 |
| 52 | M | 0.5 | Enclosure | lowland mixed and coniferous forests, meadows | August | 750 |
| 53 | F | 1 | Enclosure | lowland mixed and coniferous forests, meadows | August | 750 |
| 54 | F | 1 | Enclosure | lowland mixed and coniferous forests, meadows | August | 750 |
| 55 | M | 1 | Enclosure | lowland mixed and coniferous forests, meadows | August | 750 |
| 56 | F | 1 | Enclosure | lowland mixed and coniferous forests, meadows | August | 750 |
| 57 | F | 1 | Enclosure | lowland mixed and coniferous forests, meadows | August | 750 |
| 58 | M | 0.5 | Enclosure | lowland mixed and coniferous forests, meadows | August | 750 |
| 59 | M | 2 | Enclosure | lowland mixed and coniferous forests, meadows | September | 750 |
| 60 | M | 0.5 | Enclosure | meadow, single trees and oak groves | January | 200 |
| 61 | M | 0.5 | Enclosure | meadow, single trees and oak groves | January | 200 |
| 62 | M | 1 | Enclosure | meadow, single trees and oak groves | February | 200 |
| 63 | M | 1.5 | Enclosure | meadow, single trees and oak groves | February | 200 |
| 64 | M | 2 | Enclosure | meadow, single trees and oak groves | February | 200 |
| 65 | M | 1.5 | Enclosure | lowland mixed and coniferous forests, meadows | December | 20 |
| 66 | F | 2.5 | Enclosure | lowland mixed and coniferous forests, meadows | December | 20 |
| 67 | M | 4 | Enclosure | lowland mixed and coniferous forests, meadows | October | 20 |
| 68 | M | 2 | Enclosure | meadow, single trees and groves | May | 1 |
| 69 | F | 2 | Enclosure | meadow, single trees | December | 0.3 |
| 70 | F | 2 | Enclosure | meadow, single trees | December | 0.3 |
| 71 | F | 2 | Enclosure | meadow, single trees | November | 0.3 |
| 72* | M | 3 | Enclosure | meadow, single trees | November | 0.3 |
| 73 | F | 3 | Enclosure | meadow, single trees | November | 0.3 |
| 74 | F | 4 | Enclosure | degraded lowland mixed and coniferous forests, meadows | December | 50 |
| 75 | F | 2.5 | Enclosure | degraded lowland mixed and coniferous forests, meadows | December | 50 |
| 76 | M | 11 | Enclosure | degraded lowland mixed and coniferous forests, meadows | April | 50 |
| 77 | M | 13 | Enclosure | degraded lowland mixed and coniferous forests, meadows | September | 50 |
| 78 | M | 11 | Enclosure | degraded lowland mixed and coniferous forests, meadows | September | 50 |
| 79 | F | 1 | Enclosure | sands, pine grove | November | 0.5 |
