# Supplementary material for: Determination of hematological and biochemical values blood parameters for European bison (Bison bonasus)
Source: PLoS One. 2024 May 15;19(5):e0303457. doi: 10.1371/journal.pone.0303457 (PMC11095690; doi:10.1371/journal.pone.0303457)
Supplement: S2 Table — (DOCX) [file pone.0303457.s002.docx]

2A Table. Effect of age and sex on hematology blood parameters of European bison without diagnosed disease symptoms and raw data on hematology blood parameters of European bison used in Figures 1 and 2.

S2A Table. Effect of age and sex on hematology blood parameters of European bison without diagnosed disease symptoms (*reference category; ** in table Wald Χ^2^ test or t test was given depending on the model used: generalized linear model (GzLM) or analysis of variance (ANOVA) respectively; *** marginal means of given parameter for given age or sex group – only statistically significant variables, for measurement units see methods).

| Blood parameter | Source | *B* | *SE* | *Wald Χ^2^/t*** | *p* | *Mean (±SE) **** |
| --- | --- | --- | --- | --- | --- | --- |
| WBC (ANOVA) | Intercept | 4.916 | 0.571 | 8.608 | <0.001 |  |
|  | Age (Young) | 1.981 | 0.608 | 3.261 | 0.002 | 7.229 (0.428) |
|  | Age (Adult) | 0* |  |  |  | 5.248 (0.409) |
|  | Sex (F) | 0.663 | 0.608 | 1.091 | 0.281 |  |
|  | Sex (M) | 0* |  |  |  |  |
|  |  |  |  |  |  |  |
| RBC (ANOVA) | Intercept | 6.596 | 0.432 | 15.278 | <0.001 |  |
|  | Age (Young) | 0.520 | 0.453 | 1.149 | 0.256 |  |
|  | Age (Adult) | 0* |  |  |  |  |
|  | Sex (F) | -0.423 | 0.454 | -0.932 | 0.356 |  |
|  | Sex (M) | 0* |  |  |  |  |
|  |  |  |  |  |  |  |
| Hb (ANOVA) | Intercept | 9.470 | 0.668 | 14.187 | <0.001 |  |
|  | Age (Young) | 1.214 | 0.668 | 1.819 | 0.076 |  |
|  | Age (Adult) | 0* |  |  |  |  |
|  | Sex (F) | 0.818 | 0.675 | 1.212 | 0.232 |  |
|  | Sex (M) | 0* |  |  |  |  |
|  |  |  |  |  |  |  |
| Hct (ANOVA) | Intercept | 28.038 | 1.792 | 15.650 | <0.001 |  |
|  | Age (Young) | 3.135 | 1.797 | 1.745 | 0.088 |  |
|  | Age (Adult) | 0* |  |  |  |  |
|  | Sex (F) | 2.066 | 1.812 | 1.140 | 0.261 |  |
|  | Sex (M) | 0* |  |  |  |  |
|  |  |  |  |  |  |  |
| MCV (GLzM) | Intercept | 3.905 | 0.041 | 9111.015 | <0.001 |  |
|  | Age (Young) | -0.093 | 0.043 | 4.693 | 0.031 | 46.25 (1.435) |
|  | Age (Adult) | 0* |  |  |  | 50.76 (1.460) |
|  | Sex (F) | 0.043 | 0.043 | 1.000 | 0.317 |  |
|  | Sex (M) | 0* |  |  |  |  |
|  |  |  |  |  |  |  |
| HCH (ANOVA) | Intercept | 16.643 | 0.413 | 40.345 | <0.001 |  |
|  | Age (Young) | -0.931 | 0.434 | -2.146 | 0.037 | 15.880 (0.303) |
|  | Age (Adult) | 0* |  |  |  | 16.811 (0.298) |
|  | Sex (F) | 0.337 | 0.435 | 0.776 | 0.442 |  |
|  | Sex (M) | 0* |  |  |  |  |
|  |  |  |  |  |  |  |
| HCHC (ANOVA) | Intercept | 33.605 | 0.386 | 87.154 | <0.001 |  |
|  | Age (Young) | 0.985 | 0.404 | 2.435 | 0.019 | 34.392 (0.284) |
|  | Age (Adult) | 0* |  |  |  | 33.407 (0.275) |
|  | Sex (F) | -0.396 | 0.406 | -0.977 | 0.334 |  |
|  | Sex (M) | 0* |  |  |  |  |
|  |  |  |  |  |  |  |
| PLT (GLzM) | Intercept | 5.247 | 0.021 | 59963.675 | <0.001 |  |
|  | Age (Young) | -0.047 | 0.023 | 4.015 | 0.045 | 164.00 (2.701) |
|  | Age (Adult) | 0* |  |  |  | 171.85 (2.718) |
|  | Sex (F) | -0.202 | 0.023 | 74.715 | <0.001 | 151.79 (2.477) |
|  | Sex (M) | 0* |  |  |  | 185.68 (2.964) |
|  |  |  |  |  |  |  |
| BAND (GLzM) | Intercept | -3.427 | 0.700 | 23.963 | <0.001 |  |
|  | Age (Young) | 0.070 | 0.722 | 0.009 | 0.923 |  |
|  | Age (Adult) | 0* |  |  |  |  |
|  | Sex (F) | 0.181 | 0.725 | 0.062 | 0.803 |  |
|  | Sex (M) | 0* |  |  |  |  |
|  |  |  |  |  |  |  |
| SEG (GLzM) | Intercept | 0.916 | 0.113 | 65.625 | <0.001 |  |
|  | Age (Young) | 0.391 | 0.116 | 11.267 | <0.001 | 4.05 (0.320) |
|  | Age (Adult) | 0* |  |  |  | 2.74 (0.226) |
|  | Sex (F) | 0.182 | 0.117 | 2.428 | 0.119 |  |
|  | Sex (M) | 0* |  |  |  |  |
|  |  |  |  |  |  |  |
| EOS (GLzM) | Intercept | -0.876 | 0.256 | 11.514 | <0.001 |  |
|  | Age (Young) | 0.007 | 0.276 | 0.001 | 0.981 |  |
|  | Age (Adult) | 0* |  |  |  |  |
|  | Sex (F) | 0.153 | 0.277 | 0.306 | 0.580 |  |
|  | Sex (M) | 0* |  |  |  |  |
|  |  |  |  |  |  |  |
| LYM (GLzM) | Intercept | 0.701 | 0.106 | 43.599 | <0.001 |  |
|  | Age (Young) | 0.251 | 0.112 | 5.046 | 0.025 | 2.51 (0.193) |
|  | Age (Adult) | 0* |  |  |  | 1.95 (0.152) |
|  | Sex (F) | -0.062 | 0.112 | 0.308 | 0.579 |  |
|  | Sex (M) | 0* |  |  |  |  |
|  |  |  |  |  |  |  |
| MONO (GLzM) | Intercept | -3.448 | 0.499 | 47.628 | <0.001 |  |
|  | Age (Young) | 1.023 | 0.493 | 4.316 | 0.038 | 0.12 (0.037) |
|  | Age (Adult) | 0* |  |  |  | 0.04 (0.016) |
|  | Sex (F) | 0.576 | 0.491 | 1.377 | 0.241 |  |
|  | Sex (M) | 0* |  |  |  |  |
|  |  |  |  |  |  |  |
| BASO (GLzM) | Intercept | -3.536 | 0.581 | 34.592 | <0.001 |  |
|  | Age (Young) | -0.158 | 0.629 | 0.063 | 0.801 |  |
|  | Age (Adult) | 0* |  |  |  |  |
|  | Sex (F) | 0.253 | 0.630 | 0.161 | 0.688 |  |
|  | Sex (M) | 0* |  |  |  |  |
|  |  |  |  |  |  |  |

S2B Table. Raw data on hematology blood parameters of European bison used in Figures 1 and 2 (*WBC – white blood cells, RBC - red blood cells, Hb -hemoglobin, Hct – hematocrit, MCV - mean corpuscular volume, MCH mean corpuscular Hb, MCHC - mean corpuscular Hb concentration, PLT - platelets, SEG - neutrophils segmented, BAND – neutrophils banded, MON – monocytes, EOS – eosinophils, LYM – lymphocytes).

| Parameter* | Values on hematology blood parameters |
| --- | --- |
| Figure 1. | |
| RBC (x10^9^/L) | 2.69, 3.91, 4.37, 5.07, 5.08, 5.13, 5.31, 5.32, 5.34, 5.38, 5.46, 5.49, 5.57, 5.61, 5.63, 5.67, 5.86, 5.87, 5.88, 6, 6.03, 6.06, 6.24, 6.37, 6.40, 6.41, 6.69, 6.78, 6.85, 6.92, 7.05, 7.15, 7.15, 7.31, 7.33, 7.33, 7.34, 7.51, 7.70, 7.77, 7.77, 7.79, 7.84, 7.88, 7.92, 8.04, 8.07, 8.22, 8.28, 10.73, 11.18 |
| Hb (g/dl) | 4.3, 5.5, 8.3, 8.6, 8.6, 8.8, 8.9, 8.9, 8.9, 9.2, 9.2, 9.2, 9.2, 9.5, 9.5, 9.6, 9.7, 9.7, 9.8, 10, 10.1, 10.2, 10.2, 10.3, 10.3, 10.4, 10.4, 10.5, 10.6, 10.6, 10.7, 10.9, 11.1, 11.2, 11.3, 11.5, 11.5, 11.7, 12.3, 12.4, 12.5, 12.5, 12.7, 12.9, 13, 13.4, 14, 15.8, 16.3 |
| Hct (L/L) | 12.83, 17.51, 23.78, 25.19, 25.34, 25.36, 25.62, 25.92, 26.24, 26.59, 26.76, 28.05, 28.17, 28.23, 28.45, 28.78, 28.81, 29.04, 29.18, 29.41, 29.55, 29.84, 30.33, 30.89, 30.97, 30.99, 31.07, 31.34, 31.53, 31.63, 32.58, 32.72, 32.95, 33.23, 33.71, 33.74, 34.07, 34.62, 35.04, 36.56, 36.9, 37.02, 37.03, 37.42, 37.83, 38.16, 39.92, 47.09 |
| MCV (fL) | 39, 39, 42, 42, 42, 42, 43, 43, 44, 45, 45, 45, 45, 45, 46, 46, 46, 47, 47, 47, 48, 48, 48, 48, 48, 48, 48, 48, 48, 48, 48, 48, 49, 49, 49, 50, 50, 50, 50, 51, 51, 52, 54, 54, 56, 59, 60, 60, 61, 61, 64 |
| MCH (pg) | 13.4, 13.8, 14.2, 14.3, 14.3, 14.4, 14.5, 14.7, 14.7, 14.8, 15.2, 15.2, 15.6, 15.7, 15.8, 15.8, 15.9, 15.9, 16, 16.1, 16.2, 16.2, 16.3, 16.3, 16.4, 16.4, 16.5, 16.6, 16.6, 16.7, 16.8, 16.8, 16.9, 16.9, 16.9, 17, 17, 17, 17, 17.1, 17.1, 17.2, 17.7, 18.4, 18.7, 18.9, 18.9, 19.4, 19.5, 19.5 |
| MCHC (g/L) | 30.3, 30.4, 31.2, 31.5, 31.6, 32.3, 32.4, 32.6, 32.6, 32.7, 32.9, 32.9, 33.1, 33.2, 33.2, 33.2, 33.6, 33.6, 33.6, 33.7, 33.8, 33.9, 33.9, 34, 34, 34, 34, 34.1, 34.1, 34.2, 34.3, 34.3, 34.4, 34.4, 34.6, 34.6, 34.7, 34.7, 34.7, 34.7, 34.7, 34.8, 34.9, 34.9, 35.2, 35.6, 35.7, 35.8, 36.1, 36.5, 37.4 |
| Figure 2. | |
| WBC (x10^12^/L)) | 1.88, 2.57, 2.81, 3.39, 3.46, 3.58, 3.68, 4.15, 4.44, 4.85, 4.95, 4.97, 5.07, 5.12, 5.29, 5.3, 5.32, 5.36, 5.39, 5.45, 5.75, 5.84, 5.85, 6.02, 6.18, 6.19, 6.26, 6.33, 6.4, 6.42, 6.6, 6.68, 6.72, 6.96, 6.98, 6.99, 7, 7.17, 7.71, 7.82, 7.84, 7.89, 8.1, 8.24, 9.22, 10.01, 10.19, 10.25, 12.74 |
| BAND (x10^9^/L) | 0, 0, 0, 0, 0, 0, 0, 0, 0, 0, 0, 0, 0, 0, 0, 0, 0, 0, 0, 0, 0, 0, 0, 0, 0, 0, 0, 0, 0, 0, 0, 0, 0, 0, 0.019, 0.037, 0.049, 0.051, 0.053, 0.054, 0.055, 0.067, 0.070, 0.089, 0.136, 0.192, 0.198, 0.269, 0.408 |
| SEG (x10^9^/L) | 0.73, 1.21, 1.22, 1.47, 1.66, 1.70, 2.13, 2.17, 2.25, 2.28, 2.37, 2.42, 2.44, 2.44, 2.70, 2.72, 2.73, 2.73, 2.80, 2.83, 2.84, 2.86, 2.86, 2.97, 3.21, 3.24, 3.36, 3.49, 3.50, 3.60, 3.68, 3.71, 3.77, 3.77, 3.79, 3.92, 4.30, 4.32, 4.37, 4.42, 4.44, 4.45, 5.30, 5.33, 5.83, 5.95, 6.21, 6.45, 7.26 |
| EOS (x10^9^/L) | 0, 0, 0, 0, 0.038, 0.051, 0.054, 0.059, 0.066, 0.068, 0.069, 0.117, 0.120, 0.133, 0.134, 0.140, 0.143, 0.158, 0.194, 0.213, 0.248, 0.273, 0.277, 0.306, 0.317, 0.318, 0.331, 0.348, 0.478, 0.486, 0.501, 0.506, 0.540, 0.590, 0.619, 0.627, 0.700, 0.768, 0.823, 0.824, 0.863, 0.870, 0.892, 1.001, 1.027, 1.173, 1.360, 1.603, 1.748 |
| LYM (x10^9^/L) | 0.60, 0.99, 1.02, 1.11, 1.12, 1.18, 1.19, 1.20, 1.39, 1.51, 1.54, 1.55, 1.56, 1.62, 1.62, 1.73, 1.76, 1.79, 1.79, 1.81, 1.88, 1.93, 1.95, 1.95, 2.03, 2.07, 2.12, 2.20, 2.31, 2.33, 2.36, 2.41, 2.58, 2.58, 2.64, 2.72, 2.76, 2.77, 2.79, 2.87, 3.07, 3.16, 3.21, 3.30, 3.36, 3.55, 4.08, 4.10, 4.18 |
| BASO (x10^9^/L) | 0, 0, 0, 0, 0, 0, 0, 0, 0, 0, 0, 0, 0, 0, 0, 0, 0, 0, 0, 0, 0, 0, 0, 0, 0, 0, 0, 0, 0, 0, 0, 0, 0.051, 0.055, 0.058, 0.062, 0.062, 0.064, 0.070, 0.070, 0.077, 0.078, 0.106, 0.107, 0.107, 0.134, 0.140, 0.165, 0.255 |
| MONO (x10^9^/L) | 0, 0, 0, 0, 0, 0, 0, 0, 0, 0, 0, 0, 0, 0, 0, 0, 0, 0, 0, 0, 0, 0, 0, 0.044, 0.056, 0.058, 0.058, 0.062, 0.070, 0.077, 0.101, 0.117, 0.125, 0.127, 0.128, 0.134, 0.138, 0.140, 0.140, 0.158, 0.159, 0.162, 0.165, 0.166, 0.198, 0.205, 0.255, 0.273, 0.601 |
| PLT (x10^9^/L) | 28, 48, 54, 58, 82, 92, 93, 98, 99, 100, 106, 108, 112, 116, 120, 125, 131, 131, 132, 132, 132, 136, 139, 140, 146, 149, 157, 159, 168, 169, 169, 190, 191, 198, 204, 217, 218, 219, 225, 233, 237, 256, 261, 274, 276, 297, 311, 327, 365, 380, 274 |
