# Supplementary material for: Determination of hematological and biochemical values blood parameters for European bison (Bison bonasus)
Source: PLoS One. 2024 May 15;19(5):e0303457. doi: 10.1371/journal.pone.0303457 (PMC11095690; doi:10.1371/journal.pone.0303457)
Supplement: S3 Table — (DOCX) [file pone.0303457.s003.docx]

S3 Table. Effect of age and sex on biochemical blood parameters of European bison without diagnosed disease symptoms in analysis of variance and raw data on biochemical blood parameters of European bison used in Figures 3, 4 and 5.

S3A Table. Effect of age and sex on biochemical blood parameters of European bison without diagnosed disease symptoms in analysis of variance (*reference category; ** marginal means of given parameter for given age or sex group – only statistically significant variables, for measurement units see methods).

| Blood parameter | Source | *B* | *SE* | *t* | *p* | *Mean (±SE) *** |
| --- | --- | --- | --- | --- | --- | --- |
| AST | Intercept | 71.866 | 3.300 | 21.776 | <0.001 |  |
|  | Age (Young) | -0.094 | 3.741 | -0.025 | 0.980 |  |
|  | Age (Adult) | 0* |  |  |  |  |
|  | Sex (F) | -0.344 | 3.741 | -0.092 | 0.927 |  |
|  | Sex (M) | 0* |  |  |  |  |
|  |  |  |  |  |  |  |
| ALT | Intercept | 24.593 | 1.403 | 17.531 | <0.001 |  |
|  | Age (Young) | 2.113 | 1.572 | 1.344 | 0.183 |  |
|  | Age (Adult) | 0* |  |  |  |  |
|  | Sex (F) | 0.443 | 1.574 | 0.281 | 0.779 |  |
|  | Sex (M) | 0* |  |  |  |  |
|  |  |  |  |  |  |  |
| ALP | Intercept | 47.661 | 2.279 | 20.914 | <0.001 |  |
|  | Age (Young) | 8.683 | 2.622 | 3.312 | 0.001 | 52.524 (1.845) |
|  | Age (Adult) | 0* |  |  |  | 43.842 (1.862) |
|  | Sex (F) | -7.639 | 2.628 | -2.907 | 0.005 | 44.363 (1.792) |
|  | Sex (M) | 0* |  |  |  | 52.002 (1.918) |
|  |  |  |  |  |  |  |
| Glu | Intercept | 5.686 | 0.591 | 9.629 | <0.001 |  |
|  | Age (Young) | -0.970 | 0.668 | -1.452 | 0.151 |  |
|  | Age (Adult) | 0* |  |  |  |  |
|  | Sex (F) | 0.902 | 0.667 | 1.352 | 0.181 |  |
|  | Sex (M) | 0* |  |  |  |  |
|  |  |  |  |  |  |  |
| CREA | Intercept | 2.034 | 0.092 | 22.182 | <0.001 |  |
|  | Age (Young) | -0.229 | 0.103 | -2.219 | 0.029 | 1.726 (0.071) |
|  | Age (Adult) | 0* |  |  |  | 1.956 (0.075) |
|  | Sex (F) | -0.158 | 0.103 | -1.526 | 0.131 |  |
|  | Sex (M) | 0* |  |  |  |  |
|  |  |  |  |  |  |  |
| UREA | Intercept | 6.400 | 0.417 | 15.353 | <0.001 |  |
|  | Age (Young) | -0.511 | 0.469 | -1.088 | 0.280 |  |
|  | Age (Adult) | 0* |  |  |  |  |
|  | Sex (F) | 0.324 | 0.469 | 0.691 | 0.492 |  |
|  | Sex (M) | 0* |  |  |  |  |
|  |  |  |  |  |  |  |
| TP | Intercept | 62.760 | 1.203 | 52.174 | <0.001 |  |
|  | Age (Young) | -5.259 | 1.348 | -3.902 | <0.001 | 57.439 (0.936) |
|  | Age (Adult) | 0* |  |  |  | 62.698 (0.971) |
|  | Sex (F) | -0.124 | 1.349 | -0.092 | 0.927 |  |
|  | Sex (M) | 0* |  |  |  |  |
|  |  |  |  |  |  |  |
| Ca | Intercept | 2.330 | 0.048 | 48.180 | <0.001 |  |
|  | Age (Young) | 0.057 | 0.054 | 1.047 | 0.298 |  |
|  | Age (Adult) | 0* |  |  |  |  |
|  | Sex (F) | -0.054 | 0.054 | -0.992 | 0.324 |  |
|  | Sex (M) | 0* |  |  |  |  |
|  |  |  |  |  |  |  |
| P | Intercept | 1.483 | 0.075 | 19.893 | <0.001 |  |
|  | Age (Young) | 0.934 | 0.260 | 3.595 | <0.001 | 1.626 (0.059) |
|  | Age (Adult) | 0* |  |  |  | 1.325 (0.060) |
|  | Sex (F) | -0.983 | 0.261 | -3.772 | <0.001 | 1.317 (0.057) |
|  | Sex (M) | 0* |  |  |  | 1.634 (0.062) |
|  |  |  |  |  |  |  |
| Mg | Intercept | 0.852 | 0.041 | 20.815 | <0.001 |  |
|  | Age (Young) | 0.010 | 0.046 | 0.207 | 0.837 |  |
|  | Age (Adult) | 0* |  |  |  |  |
|  | Sex (F) | -0.010 | 0.046 | -0.221 | 0.825 |  |
|  | Sex (M) | 0* |  |  |  |  |
|  |  |  |  |  |  |  |
| K | Intercept | 50.956 | 2.046 | 24.907 | <0.001 |  |
|  | Age (Young) | 3.126 | 2.265 | 1.383 | 0.171 |  |
|  | Age (Adult) | 0* |  |  |  |  |
|  | Sex (F) | -4.548 | 2.265 | -2.008 | 0.048 | 47.97 (1.539) |
|  | Sex (M) | 0* |  |  |  | 52.52 (1.663) |
|  |  |  |  |  |  |  |
| Na | Intercept | 1369.656 | 6.991 | 195.904 | <0.001 |  |
|  | Age (Young) | -16.856 | 7.871 | -2.142 | 0.035 | 1355.71 (5.427) |
|  | Age (Adult) | 0* |  |  |  | 1372.57 (5.706) |
|  | Sex (F) | 5.828 | 7.871 | 0.740 | 0.461 |  |
|  | Sex (M) | 0* |  |  |  |  |
|  |  |  |  |  |  |  |
| Cl | Intercept | 1007.272 | 8.477 | 118.824 | <0.001 |  |
|  | Age (Young) | -7.388 | 9.758 | -0.757 | 0.452 |  |
|  | Age (Adult) | 0* |  |  |  |  |
|  | Sex (F) | 16.574 | 9.808 | 1.690 | 0.096 |  |
|  | Sex (M) | 0* |  |  |  |  |
|  |  |  |  |  |  |  |

S3B Table. Raw data on biochemical blood parameters of European bison used in Figures 3, 4 and 5 (* AST - aspartate aminotransferase, ALT - alanine aminotransferase, ALP - alkaline phosphatase, Glu - glucose, CREA - creatinine, UREA - urea, TP - total protein, Ca - calcium, P - phosphorus, Mg -magnesium, Na -sodium, K -potassium, and Cl -chlorine).

| Parameter* | Values on hematology blood parameters |
| --- | --- |
| Figure 3. | |
| AST (U/L) | 23, 38, 38.7, 46.3, 47.8, 48.2, 49.8, 50.9, 52, 52.1, 53, 54.2, 56.3, 59.6, 60.7, 61.4, 62.7, 63, 64.1, 64.5, 64.8, 65.9, 67.9, 68.3, 68.5, 69.6, 69.9, 69.9, 70.1, 70.9, 71.1, 71.1, 71.2, 71.4, 71.6, 72.5, 73.8, 73.8, 74, 74.5, 74.6, 74.6, 75, 75.4, 76.2, 78.1, 78.3, 78.5, 79.1, 79.2, 79.5, 79.5, 79.6, 80.6, 81.5, 82, 82.3, 82.6, 84.1, 84.5, 85.2, 85.7, 86.3, 86.9, 88.3, 89.3, 91.4, 92.5, 93.4, 97.3, 104.2, 115.3 |
| ALT (U/L) | 10.4, 14.3, 14.7, 15.4, 16.1, 16.8, 17, 17, 17.1, 18, 18.4, 19.8, 19.8, 20.1, 20.2, 20.5, 20.5, 20.6, 20.9, 20.9, 21, 21.6, 21.8, 22, 22.1, 22.5, 22.7, 22.8, 22.8, 23.3, 23.4, 23.5, 23.7, 24.2, 24.9, 25.2, 25.5, 25.7, 25.8, 25.9, 25.9, 26.2, 26.2, 26.6, 26.8, 27, 27.1, 27.1, 27.7, 27.9, 27.9, 27.9, 28, 28.1, 28.6, 28.7, 29.1, 29.2, 29.3, 29.4, 29.5, 29.6, 30.1, 30.2, 30.3, 30.7, 31.3, 31.4, 31.7, 32.6, 32.7, 33.2, 35.9, 40.3, 40.4, 42.2, 43, 44.7, 44.7 |
| ALP (U/L) | 23.5, 26.1, 27.6, 28.8, 32.7, 33.3, 33.8, 34.4, 34.7, 35.4, 35.9, 37.2, 38.7, 38.8, 38.9, 39.2, 39.5, 40.1, 40.9, 41.4, 41.4, 41.6, 41.7, 41.7, 42.4, 42.4, 42.6, 42.7, 42.8, 43.2, 43.2, 43.4, 44.3, 44.6, 44.9, 45.1, 45.4, 45.4, 46.5, 46.6, 48.6, 48.8, 49.5, 49.7, 49.9, 50.8, 51.3, 51.5, 52, 52, 52.6, 53.2, 53.7, 53.8, 53.8, 55.5, 55.7, 55.7, 56.2, 56.3, 56.9, 58.4, 58.5, 60.1, 63.1, 63.2, 65.4, 65.7, 67.3, 69.7, 81.8, 83.5, 85.6 |
| Figure 4. |  |
| Glu (mmol/L) | 8.7, 19.3, 27.6, 27.7, 34.7, 38.8, 45.1, 45.3, 46.2, 46.8, 49.7, 50.5, 51.5, 52.8, 52.9, 53.1, 56, 56.7, 61.4, 64.9, 65.2, 66.6, 67.4, 67.9, 68.5, 68.9, 70.3, 71.6, 72.1, 72.5, 75.9, 76.2, 76.8, 77.8, 81.5, 82.9, 85.4, 89, 91.7, 92.7, 93.9, 95.6, 101.2, 101.8, 107.5, 107.6, 109.4, 111.6, 111.8, 112, 112.6, 114.6, 117.3, 119.4, 124.4, 127.8, 130.9, 138.6, 146.4, 146.6, 151.4, 153.2, 153.3, 154.9, 157.5, 158.1, 158.4, 169.5, 173.7, 174.4, 179.4, 181, 185.2, 215.4, 220, 223.7, 264.1 |
| UREA (mmol/L) | 13.3, 14.2, 15.7, 17.1, 18.2, 18.3, 19, 20.6, 20.8, 20.8, 21.4, 21.6, 22, 24.2, 26, 27.7, 28.2, 28.5, 29.6, 29.8, 30, 30.2, 30.2, 30.3, 30.6, 31.8, 31.8, 32.9, 33.3, 34, 34.2, 35.3, 35.8, 37, 37.3, 38.5, 39, 39, 39.1, 39.7, 40, 40.2, 41.9, 42.1, 42.6, 42.9, 43, 43.1, 43.2, 43.4, 43.6, 44.5, 44.6, 44.7, 44.8, 45.2, 45.8, 45.8, 45.9, 46.5, 46.7, 46.8, 46.8, 46.9, 47.2, 48, 48.1, 48.5, 49, 49.3, 49.5, 50, 50.7, 56.6, 64.3, 64.5, 67.2, 68.5 |
| CREA (mg/%) | 0.5, 0.83, 1, 1.03, 1.05, 1.11, 1.17, 1.29, 1.31, 1.41, 1.43, 1.44, 1.46, 1.49, 1.49, 1.5, 1.53, 1.54, 1.54, 1.55, 1.59, 1.6, 1.61, 1.62, 1.63, 1.64, 1.65, 1.66, 1.66, 1.71, 1.73, 1.73, 1.74, 1.75, 1.75, 1.75, 1.76, 1.78, 1.78, 1.81, 1.82, 1.86, 1.86, 1.87, 1.89, 1.9, 1.91, 1.91, 1.92, 1.93, 1.94, 1.94, 1.95, 1.99, 2, 2.01, 2.01, 2.05, 2.08, 2.09, 2.12, 2.12, 2.16, 2.16, 2.29, 2.32, 2.34, 2.35, 2.37, 2.37, 2.4, 2.48, 2.5, 2.56, 2.71, 2.79, 3, 3.24 |
| TP (g/L) | 40.9, 41.5, 48.4, 50.8, 51.7, 52.3, 52.3, 53.1, 53.1, 53.2, 53.3, 54.3, 54.5, 54.9, 55, 55.6, 55.7, 55.8, 55.9, 55.9, 56.3, 56.6, 56.9, 56.9, 57.4, 57.6, 57.7, 57.7, 57.8, 57.8, 58.1, 58.3, 58.6, 58.8, 59, 59.3, 59.4, 59.5, 59.7, 59.8, 59.8, 59.9, 59.9, 60, 60.2, 60.3, 60.6, 60.7, 60.9, 61.1, 61.2, 61.3, 61.5, 61.7, 62.1, 62.2, 62.9, 63.1, 63.2, 63.7, 64, 64.2, 64.6, 65, 65.6, 65.6, 65.9, 65.9, 66.8, 67.6, 67.6, 68.4, 69.1, 69.7, 70.7, 72.6, 74.2, 74.4, 77.6 |
| Figure 5. | |
| Ca (mmol/L) | 7.37, 7.4, 7.58, 7.82, 7.86, 8.01, 8.04, 8.06, 8.07, 8.12, 8.13, 8.14, 8.28, 8.3, 8.32, 8.36, 8.4, 8.43, 8.47, 8.51, 8.53, 8.55, 8.59, 8.61, 8.65, 8.9, 8.91, 8.92, 9, 9.07, 9.12, 9.13, 9.21, 9.27, 9.27, 9.3, 9.354, 9.39, 9.4, 9.47, 9.48, 9.49, 9.5, 9.52, 9.55, 9.58, 9.58, 9.61, 9.64, 9.66, 9.66, 9.7, 9.77, 9.78, 9.8, 9.8, 9.82, 9.88, 9.89, 9.97, 10.05, 10.1, 10.1, 10.1, 10.24, 10.3, 10.3, 10.31, 10.37, 10.4, 10.44, 10.48, 10.7, 10.8, 10.8, 10.8, 11.1, 11.5, 11.7 |
| Mg (mmol/L) | 1.02, 1.2, 1.23, 1.24, 1.37, 1.38, 1.38, 1.41, 1.43, 1.44, 1.54, 1.57, 1.59, 1.6, 1.61, 1.64, 1.67, 1.7, 1.75, 1.8, 1.8, 1.81, 1.81, 1.81, 1.83, 1.87, 1.88, 1.88, 1.9, 1.91, 1.91, 1.92, 1.96, 2, 2, 2.02, 2.03, 2.04, 2.05, 2.09, 2.09, 2.12, 2.12, 2.14, 2.15, 2.16, 2.19, 2.2, 2.2, 2.21, 2.25, 2.25, 2.25, 2.27, 2.27, 2.27, 2.29, 2.32, 2.33, 2.37, 2.38, 2.39, 2.41, 2.42, 2.45, 2.49, 2.49, 2.56, 2.61, 2.64, 2.66, 2.81, 2.81, 2.81, 2.87, 3.02, 3.42, 3.55 |
| Na (mmol/L) | 127.9, 129, 129.7, 129.8, 130.1, 130.5, 130.5, 131.9, 132.2, 132.6, 132.7, 133, 133.3, 133.7, 133.7, 133.7, 134, 134.1, 134.1, 134.1, 134.1, 134.1, 134.1, 134.4, 134.4, 134.4, 134.4, 134.8, 134.8, 135.1, 135.2, 135.9, 136.3, 136.3, 136.3, 136.3, 136.3, 136.8, 136.9, 136.9, 136.9, 137, 137.2, 137.2, 137.2, 137.4, 137.4, 137.4, 137.4, 137.4, 137.4, 137.6, 137.8, 138.2, 138.2, 138.2, 138.2, 138.6, 138.6, 138.9, 139, 139.2, 139.3, 139.3, 139.3, 139.5, 139.7, 139.9, 140.1, 140.1, 140.2, 140.5, 140.8, 142, 143.2, 143.2, 144.4, 145.8 |
| P (mmol/L) | 1.31, 1.6, 1.76, 2.26, 2.42, 2.42, 2.49, 2.92, 2.93, 3.14, 3.25, 3.34, 3.39, 3.52, 3.58, 3.69, 3.76, 3.8, 3.87, 3.88, 3.88, 3.94, 3.95, 3.95, 3.96, 3.98, 4.06, 4.09, 4.15, 4.16, 4.22, 4.22, 4.23, 4.27, 4.27, 4.3, 4.31, 4.33, 4.41, 4.42, 4.45, 4.51, 4.52, 4.62, 4.65, 4.68, 4.69, 4.72, 4.77, 4.83, 4.87, 4.87, 4.98, 5.02, 5.08, 5.11, 5.39, 5.47, 5.51, 5.59, 5.67, 5.79, 5.82, 5.84, 5.86, 5.89, 5.94, 5.97, 6.14, 6.18, 6.2, 6.4, 6.44, 6.94, 7.01, 7.21, 7.22, 7.44 |
| K (mmol/L) | 3.36, 3.56, 3.66, 3.67, 3.75, 3.76, 3.8, 3.86, 3.89, 3.91, 3.98, 4, 4.05, 4.09, 4.13, 4.15, 4.15, 4.19, 4.21, 4.22, 4.23, 4.24, 4.27, 4.32, 4.33, 4.33, 4.34, 4.37, 4.53, 4.58, 4.58, 4.6, 4.61, 4.62, 4.66, 4.68, 4.7, 4.82, 4.86, 4.87, 4.88, 4.91, 4.94, 5, 5.01, 5.06, 5.07, 5.11, 5.14, 5.2, 5.21, 5.21, 5.25, 5.28, 5.31, 5.36, 5.51, 5.67, 5.67, 5.8, 5.81, 5.82, 5.85, 6.14, 6.21, 6.22, 6.24, 6.36, 6.43, 6.47, 6.59, 6.59, 6.68, 6.71, 6.78, 7.09, 7.56, 8.1 |
| Cl (mmol/L) | 93.4, 93.9, 95.1, 95.3, 95.4, 95.5, 95.8, 96.4, 96.5, 96.7, 97.1, 97.9, 98.1, 98.2, 98.6, 98.6, 98.6, 98.6, 98.6, 98.6, 98.6, 98.9, 99.4, 99.7, 99.7, 99.7, 99.7, 100, 100.1, 100.1, 100.2, 100.4, 100.4, 100.4, 100.5, 100.5, 100.7, 100.9, 101.2, 101.2, 101.5, 101.9, 102.3, 102.3, 102.3, 102.6, 102.7, 102.7, 102.8, 103.3, 103.5, 103.8, 104.2, 104.4, 104.9, 105, 105.1, 105.6, 105.7, 105.7, 106.2, 106.4, 106.7, 106.7, 107.2, 110.1, 110.3, 110.6, 111.6 |
